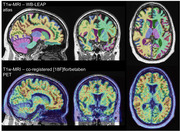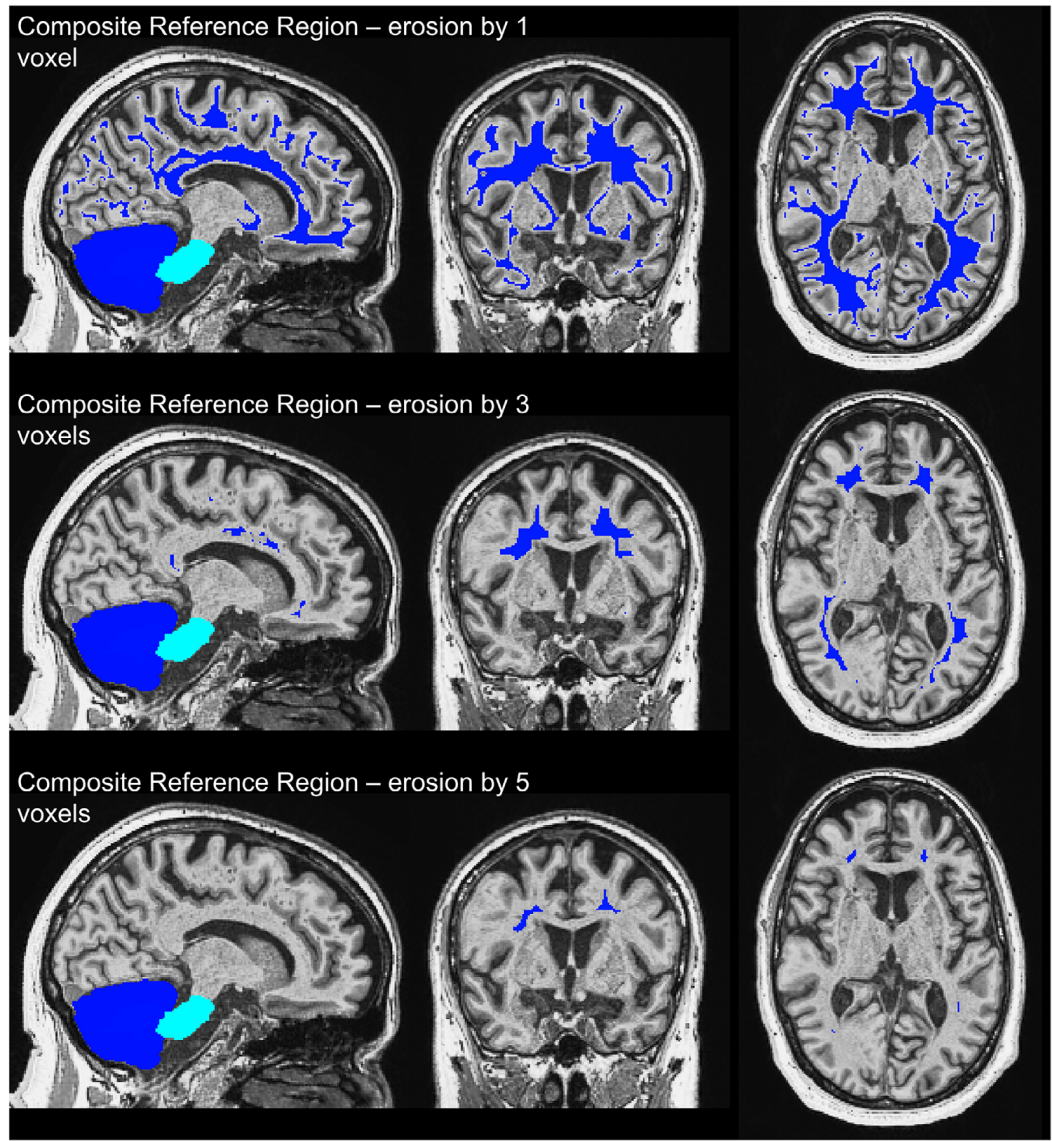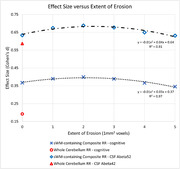# Erosion of The Supratentorial White Matter Reference for Increased Power in Longitudinal Amyloid PET

**DOI:** 10.1002/alz.086084

**Published:** 2025-01-09

**Authors:** Colm J McGinnity, Luis R Peraza, Richard G Parker, Robin Wolz

**Affiliations:** ^1^ IXICO, London UK; ^2^ IXICO, London, Greater London UK

## Abstract

**Background:**

Use of the supratentorial white matter (sWM) as a reference region for amyloid PET, alone or in a composite, can facilitate the detection of subtle changes in amyloid load (Wong *et al.*, 2010; Landau *et al.*, 2015). Investigators often erode the sWM labels to reduce the influence of signal “spill‐in” from grey matter, but the optimal extent of erosion is unclear, and evaluations for ^18^F tracers other than [^18^F]florbetapir are scarce (Bullich *et al.*, 2017). We aimed to determine the optimal extent of erosion of the supratentorial white matter by the ability to differentiate between participants who experience divergent disease trajectories.

**Method:**

We used longitudinal PET and T1w‐MR images acquired from participants who did not have Alzheimer’s disease at baseline (sources: ADNI https://adni.loni.usc.edu/, AMYPAD https://amypad.eu/). The participants were classified as “*Decline*” or “*Stable*” according to change in cognitive measures, and alternatively in cerebrospinal fluid amyloid‐β42 (CSF‐Aβ42; where available). We used IXICO’s *MultiLEAP* pipeline to automatically segment the T1w‐MRI into 143 regions with high accuracy (Figure 1). Standardised uptake value ratios (SUVRs) were calculated for the global cortical region using the whole cerebellum (WC) reference region, and using pons‐eroded sWM‐WC composites after erosion by 0 – 5 (1 mm3) voxels (Figure 2). We compared effect sizes (ΔSUVR/year – *Decline* versus *Stable*) across all reference regions.

**Result:**

In preliminary analyses using [^18^F]florbetaben data (scan interval 3±1 years), the WC reference region yielded Cohen’s d of 0.19 for 112 participants classified via cognitive measures, and 0.59 for 35 who could be classified via CSF‐Aβ42. The sWM‐containing composites yielded d values that were 1.8 – 2.1 and 1.08 – 1.17 times higher, respectively, depending on extent of erosion (Figure 3). For both classifications, erosion by two voxels yielded the maximal d (0.40 and 0.69) – equivalent to mean increases in SUVR of 0.8%/1.3% and 0.3%/‐0.3% per year for *Decline* and *Stable*, respectively.

**Conclusion:**

Eroded sWM‐containing reference regions appear to offer greater sensitivity to longitudinal change than the WC. Validation of this finding in a larger population and for additional radiotracers is ongoing. We will examine the influence of scanner model on the optimal extent of erosion.